# Traumatic Tetanus and Death from Vaccination

**Published:** 1882-11

**Authors:** 


### Traumatic Tetanus and Death from Vaccination.

Dr. Bates, of Columbia, reported a case of tetanus from vaccination, at the meeting of the South Carolina Medical Association (*Medical News*). Ben. Jones, a mulatto, was vaccinated, Feb. 9th, on the arm, with carefully-selected humanized virus. He was again seen March 8th, when he had ordinary symptoms of tetanus. Was examined next day by Drs. Talley and Howe. A most careful inquiry into the history of the case, and a searching examination of the body, revealed nothing to cause it, except a small, healthy-looking, painless ulcer at the spot where vaccination had been performed a month before. The disease advanced and caused death in fifteen days, in spite of careful treatment.

